# Differential genetic interactions between Sgs1, DNA-damage checkpoint components and DNA repair factors in the maintenance of chromosome stability

**DOI:** 10.1186/2041-9414-2-8

**Published:** 2011-10-31

**Authors:** Lillian Doerfler, Lorena Harris, Emilie Viebranz, Kristina H Schmidt

**Affiliations:** 1 Department of Cell Biology, Microbiology and Molecular Biology, University of South Florida, 4202 E. Fowler Avenue, Tampa, FL 33620, USA

**Keywords:** genome instability, translocations, Sgs1, mitotic recombination, DNA-damage checkpoint

## Abstract

**Background:**

Genome instability is associated with human cancers and chromosome breakage syndromes, including Bloom's syndrome, caused by inactivation of BLM helicase. Numerous mutations that lead to genome instability are known, yet how they interact genetically is poorly understood.

**Results:**

We show that spontaneous translocations that arise by nonallelic homologous recombination in DNA-damage-checkpoint-defective yeast lacking the BLM-related Sgs1 helicase (*sgs1Δ mec3Δ*) are inhibited if cells lack Mec1/ATR kinase. Tel1/ATM, in contrast, acts as a suppressor independently of Mec3 and Sgs1. Translocations are also inhibited in cells lacking Dun1 kinase, but not in cells defective in a parallel checkpoint branch defined by Chk1 kinase. While we had previously shown that *RAD51 *deletion did not inhibit translocation formation, *RAD59 *deletion led to inhibition comparable to the *rad52Δ *mutation. A candidate screen of other DNA metabolic factors identified Exo1 as a strong suppressor of chromosomal rearrangements in the *sgs1Δ *mutant, becoming even more important for chromosomal stability upon *MEC3 *deletion. We determined that the C-terminal third of Exo1, harboring mismatch repair protein binding sites and phosphorylation sites, is dispensable for Exo1's roles in chromosomal rearrangement suppression, mutation avoidance and resistance to DNA-damaging agents.

**Conclusions:**

Our findings suggest that translocations between related genes can form by Rad59-dependent, Rad51-independent homologous recombination, which is independently suppressed by Sgs1, Tel1, Mec3 and Exo1 but promoted by Dun1 and the telomerase-inhibitor Mec1. We propose a model for the functional interaction between mitotic recombination and the DNA-damage checkpoint in the suppression of chromosomal rearrangements in *sgs1Δ *cells.

## Background

Eukaryotic cells have mechanisms at their disposal for the detection and repair of spontaneous and induced DNA lesions, thus preventing them from giving rise to potentially abnormal daughter cells. However, if these mechanisms are defective or overwhelmed by damage, deleterious chromosomal rearrangements can arise. A multitude of genes and genetic pathways for the maintenance of genome stability has been identified mostly using genetic screens in simple model organisms such as the yeast *Saccharomyces cerevisiae*. They include DNA damage checkpoints, DNA repair factors and proteins for processing of recombination substrates and intermediates [1-10]. The importance of the same mechanisms for maintaining genome stability in human cells is highlighted by the association of mutations in the human homologues of these yeast genes with chromosome breakage syndromes, which are characterized by signs of premature aging and/or cancer development. The syndromes include Nijmegen breakage syndrome associated with mutations in *NBS1*, the homologue of yeast *XRS2 *[11-13]; Bloom's syndrome and Werner syndrome associated with mutations in *BLM *and *WRN *, respectively, both related to yeast *SGS1 *[14,15]; and ataxia telangiectasia associated with mutations in *ATM *[16], which is related to yeast *TEL1 *[17].

Yeast *SGS1 *encodes a 5' to 3' DNA helicase that preferentially unwinds three- and four-way junctions typical of replication and recombination intermediates and has recently been shown to collaborate with Exo1 in the long-range processing of double-strand breaks (DBSs) [18-21]. Without Sgs1, cells accumulate gross-chromosomal rearrangements (GCRs), exhibit elevated levels of mitotic recombination, have a reduced replicative lifespan and are sensitive to chemicals that alkylate DNA or slow replication forks [2,22-26]. Among DNA-damage checkpoint components, Mec1 kinase, also considered the homolog of mammalian ATR [27-29], has been identified as one of the strongest suppressors of GCRs in yeast [3,4]. Other cellular phenotypes of *mec1*Δ mutants include increased sensitivity to DNA damaging agents and deficient DNA-damage checkpoint response [30], instability of stalled forks [31], accumulation of DNA breaks [32] and, in addition to these mitotic defects, deficiencies in meiotic checkpoint activation and recombination [33-35]. In contrast to Mec1, cells lacking the Tel1 checkpoint kinase, which is related to mammalian ATM [17,36], are not sensitive to DNA damaging agents [17], do not accumulate GCRs above wildtype levels [3], but show telomere erosion [36]. Synergistic interactions between *mec1Δ *and *tel1*Δ mutations have been reported for many phenotypes, suggesting a functional relationship and redundancy between the two kinases [3,17,37,38]. Other checkpoint components, such as those involved in sensing DNA damage (Mec3, Rad24), appear to have only small to moderate roles in suppressing GCRs in yeast [3,4]. In cells lacking the Sgs1 helicase, however, Mec3 and Rad24 strongly suppress overall genome instability [3,4] as well as the formation of spontaneous, recurring translocations between short identical sequences in non-allelic, but related, DNA sequences [10]. Utilizing the high susceptibility of the *sgs1Δ mec3Δ *mutant to recurring translocation formation between *CAN1, LYP1 *and *ALP1*, we have in the current study conducted a candidate screen to identify two types of DNA metabolic factors - those that are required for the formation of recurring translocations in the *sgs1Δ mec3Δ *mutant and those that act independently of Sgs1 and Mec3 to suppress translocations. For this purpose, *mec1Δ, tel1Δ, dun1Δ, chk1Δ *and *rad59Δ *mutations were introduced into the *sgs1Δ mec3Δ *mutant and the accumulation of recurring translocations was assessed. We further determined how the lack of other DNA metabolic factors (*yen1Δ, lig4Δ, exo1Δ, rad1Δ, pol32Δ*) affects the accumulation of genome rearrangements, identifying a strong synergistic interaction between *sgs1Δ *and *exo1Δ*. We propose an integrated model for independent, functional interactions between Sgs1, HR subpathways and various DNA-damage-checkpoint branches in the suppression of chromosomal rearrangements.

## Results and discussion

### Functional interaction between Sgs1 and DNA-damage checkpoint components Mec3, Mec1, Tel1, Dun1 and Chk1 in the suppression of chromosomal translocations

Chromosomal translocations between short stretches of homology in nonallelic sequences that are naturally present in the yeast genome, such as the highly similar, but diverged *CAN1 *(on chromosome V), *ALP1 *and *LYP1 *genes (on chromosome XIV, 60-65% identity), are normally suppressed in yeast. However, they are recurrent in *sgs1Δ *mutants with certain additional DNA-metabolic defects, including *mec3Δ, rad24Δ, cac1Δ, asf1Δ *and *rfc5-1 *[10]. One of the mutants most susceptible to recurring translocations between the *CAN1, LYP1 *and *ALP1 *loci is the *sgs1Δ mec3Δ *mutant, whereas translocations are not found in the *sgs1Δ mec1Δ *mutant [10]. Here, we wanted to test whether the lack of *CAN1/LYP1/ALP1 *translocations in the *sgs1Δ mec1Δ *mutant meant that Mec1 was not a suppressor of translocations and therefore its deletion had no affect on translocation formation, or that Mec1 was actually required for the formation of viable chromosomal translocations. If the latter was true, we expected that introducing a *mec1Δ *mutation into the highly susceptible *sgs1Δ mec3Δ *strain should inhibit the accumulation *CAN1/LYP1/ALP1 *translocations. Indeed, we found that while deleting *MEC1 *led to a synergistic increase (~ 7-fold) in the rate of all GCR types compared to the *sgs1Δ mec3Δ *mutant (P < 0.0001), screening of GCR clones obtained from 431 individual *sgs1Δ mec3Δ mec1Δ *cultures failed to reveal a single *CAN1/LYP1/ALP1 *translocation, signifying a > 7-fold decrease in the translocation rate compared to the *sgs1Δ mec3Δ *mutant (Table 1). The synergistic GCR rate increase in the *sgs1*Δ *mec3Δ mec1Δ *mutant shows that Mec1 can activate its targets through Mec3-independent sensing of DNA damage. This may occur by Mec1-Ddc2 itself recognizing and binding to DNA lesions [39,40] or through DNA-damage sensors other than the Mec3 clamp signaling to Mec1. The synergistic GCR rate increase in the *sgs1Δ mec3Δ mec1Δ *mutant also indicates that the failure to form *CAN1/LYP1/ALP1 *translocations when *MEC1 *is deleted is not due to an inability to form viable GCRs, but rather suggests that DNA lesions are channeled into GCR pathways other than homology-driven translocation. Most likely, Mec1 promotes chromosomal translocations by inhibiting *de novo *telomere synthesis at chromosome breaks [1], for example by phosphorylating the telomerase-inhibitor Pif1 [41] and by phosphorylating Cdc13 and thus preventing its accumulation at DNA breaks [42]. In a haploid wildtype cell, these chromosomal translocations are expected to be rare due to restraints placed on homologous recombination events by the need for relative long regions of sequence identity. However, when the restraints on homologous recombination are relaxed and spontaneous DNA lesions are not properly detected by the DNA-damage checkpoint, as could be assumed for the *sgs1Δ mec3Δ *mutant, chromosomal translocations are promoted and occur between much shorter regions of sequence identity, such as the 5-41-bp segments present in *CAN1*, *LYP1 *and *ALP1*.

**Table 1:** Functional interaction between Sgs1 and components of the DNA-damage checkpoint in the suppression of GCRs and translocations between *CAN1, LYP 1 *and *ALP1 *genes.

	All GCR types*^a^*		*CAN1*/*LYP1*/*ALP1 *translocations*^b^*		Frequency of *CAN1/LYP1/ALP1* translocation types*^c^*		
	
Relevant Genotype*^d^*	Rate	95% CI	Rate	Frequency	*CAN1-ALP1*	*CAN1-LYP1*	*CAN1-LYP1-ALP1*
wildtype	1.1	< 1 - 6.2	ND	ND	ND	ND	ND
*sgs1*	220	144-276	< 7.3	0/30	0/30	0/30	0/30
*rad17*	57	26-74	ND	ND	ND	ND	ND
*mec3*	46	18-75	< 1.5	0/30	0/30	0/30	0/30
*mec3 rad17*	49	32-64	ND	ND	ND	ND	ND
*sgs1 rad17*	2515	903-4160	< 101	0/25	0/25	0/25	0/25
*sgs1 mec3*	1297	1120-2030	173	20/150	7/150	3/150	7/150
*sgs1 mec3 rad17*	1690	1247-2230	75	2/45	1/45	1/45	0/45
*tel1*	2	ND	ND	ND	ND	ND	ND
*tel1 mec3*	453	340-638	15	1/30	1/30	0/30	0/30
*tel1 rad17*	129	73-246	< 8.6	0/15	0/15	0/15	0/15
*sgs1 tel1*	227	46-418	ND*^b^*	ND	ND	ND	ND
*sgs1 tel1 rad17*	27600	22430-39653	4600	6/36	1/36	1/36	4/36
*sgs1 tel1 mec3*	57370	47157-76301	2674	11/236	0/236	6/236	4/236
*sgs1 tel1 mec3 rad17*	31960	23400-51800	ND	ND	ND	ND	ND
*mec1*	471	209-859	ND	ND	ND	ND	ND
*sgs1 mec1*	1930	960-2452	< 10	0/190	0/190	0/190	0/190
*sgs1 mec1 mec3*	9628	5870-12100	< 22	0/431	0/431	0/431	0/431
*chk1*	42	25-132	ND	ND	ND	ND	ND
*sgs1 chk1*	446	337-528	< 15	0/30	0/30	0/30	0/30
*sgs1 chk1 mec3*	1099	725-1613	147	4/30	1/30	0/30	3/30
*dun1*	252	86-472	ND	ND	ND	ND	ND
*sgs1 dun1*	1145	698-1910	< 23	0/50	0/50	0/50	0/50
*sgs1 dun1 mec3*	2800	2270-3570	< 21	0/135	0/135	0/135	0/135

*^a ^*GCR rate (Can^r ^5-FOA^r ^× 10^-10)^. 95% confidence intervals (CIs) for median GCR rates were calculated according to Nair [80], where non-overlapping confidence intervals indicate statistically significant differences between median GCR rates. GCR rates of wildtype [81], *sgs1 *[82], *mec3, sgs1 mec3 *[60], *tel1 *[3] were reported previously.

*^b ^*Rate of accumulating translocations between *CAN1, LYP1 *and/or *ALP1 *genes (x 10^-10)^. GCR clones from *sgs1, mec3, sgs1 mec3, sgs1 tel1 *and *sgs1 mec1 *were previously screened for *CAN1*/*LYP1*/*ALP1 *translocations [10,60].

*^c ^*Types of *CAN1*/*LYP1*/*ALP1 *translocations were determined by sequencing. Of the 20 *CAN1*/*LYP1*/*ALP1 *translocations identified among 150 GCR clones from the *sgs1 mec3 *mutant, 17 were identified as being either *C/A*, *C/L/A *or *C/L *translocations and 3 clones had a mixture of multiple translocations [60].

^d ^All mutants with a *mec1 *deletion also contain a *sml1 *deletion.

Deleting *TEL1*, which encodes another DNA-damage checkpoint kinase that is considered at least partially functionally redundant with Mec1, had the same effect as deleting *MEC1 *on the accumulation of all types of GCR (Table 1), as evidenced by the 44-fold increase in the overall GCR rate compared to the *sgs1Δ mec3Δ *mutant (5.7 × 10^-6 ^versus 1.3 × 10^-7^, P < 0.0001). However, deleting *TEL1 *had the opposite effect on *CAN1/LYP1/ALP1 *translocation formation (Table 1). Instead of inhibiting *CAN1/LYP1/ALP1 *translocations like the *mec1Δ *mutation, the *tel1Δ *mutation led to an increase (~15-fold) in *CAN1/LYP1/ALP1 *translocations (Table 1). Unlike *mec1Δ *mutants, mutants lacking Tel1 are impaired in their ability to maintain telomeres [36] and may thus be unable to heal DNA breaks by *de novo *telomere addition. Thus, in the absence of Tel1, DNA breaks may be channeled into alternative pathways for repair, such as HR, and more frequently give rise to *CAN1*/*LYP1*/*ALP1 *rearrangements under conditions that favor aberrant HR such as those in the *sgs1Δ mec3Δ *mutant. That failure to activate either Tel1 or Mec3-checkpoint pathways contributes independently to recurrent *CAN1/LYP1/ALP1 *translocation formation suggests that both ssDNA overhangs or gaps, thought to be sensed in a Mec3-dependent manner, and DSBs, thought to be sensed in a Tel1-dependent manner, can lead to *CAN1/LYP1/ALP1 *translocations and that they accumulate in unperturbed *sgs1Δ *cells spontaneously. The synergistic increase in overall genome instability in the *sgs1Δ mec3Δ tel1Δ *mutant might also indicate that in the absence of lesion binding by the Mec3 clamp some lesions are further processed and eventually detected by the Tel1-dependent pathway. For example, a stalled replication fork might eventually be processed into double-stranded ends in an attempt at fork restart by fork regression or template-switching.

Thus, both Tel1 and Mec1 act independently of Mec3 and Sgs1 to strongly suppress overall genome instability, but they affect *CAN1/LYP1/ALP1 *translocation formation in opposite ways. The inhibition of *CAN1*/*LYP1*/*ALP1 *translocations upon *MEC1 *deletion as opposed to their increase upon *TEL1 *deletion can most likely be explained by their opposite effects on telomere synthesis, with Mec1 inhibiting it and Tel1 promoting it. This is also consistent with the previous report of different GCR spectra in the *tel1Δ *and *mec1Δ *single mutants [1]. Apart from regulating telomere maintenance factors, it is also conceivable that the DNA-damage checkpoint-dependent phosphorylation of homologous recombination factors, such as Rad55, Slx4 and Mus81 [43-47] contributes to differential regulation of translocation formation in the *sgs1Δ mec3Δ *mutant.

The opposing effects of Tel1 and Mec1 on *CAN1/LYP1/ALP1 *translocation formation led us to investigate other DNA-damage checkpoint components in *sgs1Δ *and *sgs1Δ mec3Δ *mutants. We found that deletion of either *CHK1 *or *DUN1 *led to a synergistic increase in overall genome instability when combined with an *sgs1Δ *mutation (P < 0.0001), however only the *dun1Δ *mutation caused a further significant GCR rate increase in the *sgs1Δ mec3Δ *mutant (P < 0.0001, Table 1) whereas the *chk1Δ *mutation did not (P = 0.1615, Table 1). Analysis of the GCR types revealed accumulation of *CAN1*/*LYP1*/*ALP1 *translocations in the Chk1-deficient *sgs1Δ mec3Δ *mutant at a similar rate as in the *sgs1Δ mec3Δ *mutant, but not in the Dun1-deficient *sgs1Δ mec3Δ *mutant (Table 1), indicating that Dun1, like Mec1, promotes translocation events between *CAN1, LYP1 *and *ALP1 *whereas Chk1 does not. This is likely due to Mec1-mediated activation of Dun1 kinase, which in turn inactivates the transcription repressor Crt1, thus allowing transcription of several DNA-damage inducible genes [48,49]. Chk1 kinase is also activated through Mec1 in response to DNA damage and causes a transient G2/M arrest by blocking anaphase progression [50,51]. However, in contrast to Dun1, Chk1 is not thought to regulate DNA repair pathways, and its deletion did not inhibit translocation formation in the *sgs1Δ mec3Δ *mutant (Table 1). As expected, deletion of *RAD17*, which encodes another subunit of the Mec3/Rad17/Ddc1 checkpoint clamp, had a similar effect on *CAN1*/*LYP1*/*ALP1 *translocation formation in the *sgs1Δ tel1Δ *mutant as deletion of *MEC3 *(Table 1). The detection of a *CAN1*/*LYP1*/*ALP1 *translocation in two strains that expressed wildtype Sgs1 (*mec3Δ tel1Δ *(Table 1) and *mec3Δ tel1Δ rad17Δ (*not shown)) suggests that even in the presence of wildtype Sgs1 cells may accumulate *CAN1*/*LYP1*/*ALP1 *translocations as long as they are deficient in at least two independent suppressors of translocation formation, such as Tel1 and Mec3 identified here.

### Deletion of *RAD59 *inhibits spontaneous interchromosomal translocations between short repeats

We previously showed that translocations between *CAN1*, *LYP1 *and *ALP1 *in the *sgs1Δ mec3Δ *mutant are Rad52-dependent, but translocations still formed when Rad51 was absent [10]. To assess the role of other HR factors in translocation formation we deleted *RAD59 *in the highly susceptible *sgs1Δ mec3Δ *mutant and measured the rate of accumulating all types of GCRs as well as *CAN1/LYP1/ALP1 *translocations. One *CAN1*/*LYP1*/*ALP1 *rearrangement was identified among GCR clones obtained from 158 independent cultures of the *sgs1Δ mec3Δ rad59Δ *mutant (Table 2), indicating a 10-fold reduction in the *CAN1*/*LYP1*/*ALP1 *translocation rate compared to the *sgs1Δ mec3Δ *mutant. Thus, similar to Rad52, Rad59 is required for interchromosomal translocations between short identical sequences in related genes. If Rad59 was indeed required for translocation formation, we predicted that the formation of *CAN1*/*LYP1*/*ALP1 *translocations in the *sgs1Δ mec3Δ rad51Δ *mutant would also be inhibited by a *rad59Δ *mutation. Thus, we generated an *sgs1Δ mec3Δ rad51Δ rad59Δ *mutant and screened for *CAN1*/*LYP1*/*ALP1 *translocations. Among 168 independent GCR clones we identified one *CAN1*/*LYP1*/*ALP1 *translocation, indicative of a 28-fold reduction of the translocation rate compared to the *sgs1Δ mec3Δ rad51Δ *mutant (Table 2). Thus translocations between *CAN1*, *LYP1 *and/or *ALP1 *can form through Rad52/Rad59-mediated HR that does not require Rad51. Rad59 has recently also been shown to contribute to GCRs mediated by certain Ty-elements and to translocations involving short DNA sequences of limited sequence identity [6,52].

**Table 2:** Effect of homologous recombination mutations on the ability of the *sgs1 mec3 *mutant to accumulate GCRs and form rearrangements between the *CAN1, LYP 1 *and *ALP1 *genes.

	All GCR types*^a^*		*CAN1*/*LYP1*/*ALP1* translocations*^b^*	
	
Relevant genotype	Rate	95% CI	Rate	Frequency
wildtype	1.1	< 1-6.2	ND	ND
*rad51^a^*	< 8	< 7-15	ND	ND
*rad52 *	138	16-267	ND	ND
*rad59*	24	13-50	ND	ND
*sgs1 ^a^*	220	144-276	< 7.3	0/30
*mec3 *^*a*^	46	18-75	< 1.5	0/30
*sgs1 rad59*	126	107-300	ND	ND
*sgs1 rad59 rad51*	118	49-154	ND	ND
*sgs1 mec3 ^a^*	1297	1120-2030	173	20/150
*sgs1 mec3 rad51 ^a^*	1491	ND*^c^*	198	4/30
*sgs1 mec3 rad52 ^a^*	3168	ND*^c^*	< 23	0/136
*sgs1 mec3 rad59*	2476	1595-3187	16	1/158
*sgs1 mec3 rad59 rad51*	1124	734-1460	7	1/168

*^a ^*Median rate of cells resistant to canavanine and 5-FOA (Can^r ^5-FOA^r ^× 10^-10)^. 95% confidence intervals (CIs) for median GCR rates were calculated according to Nair [80], where non-overlapping confidence intervals indicate statistically significant differences between median GCR rates. GCR rates for wildtype [81], *sgs1 *[82], *mec3, sgs1 mec3 *[60], *rad51, sgs1 mec3 rad51 *and *sgs1 mec3 rad52 *mutants [10] were reported previously and are included for comparison.

*^b ^*Rate of accumulating translocations between *CAN1, LYP1 *and/or *ALP1 *(Can^r ^5-FOA^r ^× 10^-10)^. GCR clones from *sgs1, mec3, sgs1 mec3, sgs1 mec3 rad51, sgs1 mec3 rad52 *were previously screened for *CAN1/LYP1/ALP1 *translocations [10,60]. ND, not determined.

^c ^To determine 95% CIs for *sgs1 mec3 rad51 *and *sgs1 mec3 rad52 *mutants, GCR rates were re-measured for the current study. The GCR rate for the *sgs1 mec3 rad51 *mutant was 1933 × 10^-10 ^(95% CIs: 601-2240 × 10^-10^) and the GCR rate for the *sgs1 mec3 rad52 *mutant was 2220 × 10^-10 ^(951-3470 × 10^-10^). The previously reported rates fall within the 95% CIs determined in the current study.

While Rad52 is required for all HR in yeast, some DNA breaks can be repaired by HR pathways that do not require Rad51, including single-strand annealing (SSA), break-induced replication (BIR) and recombination-mediated telomere-lengthening Type II [53-58]. SSA is a mechanism for the repair of a DSB between repeated DNA elements and requires Rad59, but not Rad51 [59]. In order for the interchromosomal *CAN1*/*LYP1*/*ALP1 *rearrangements to arise by SSA, however, at least two DSBs would have to occur in the same cell - one DSB within or downstream of *CAN1 *on chromosome V and one DSB near *ALP1 *(or *LYP1*) on chromosome XIV. Resection would expose the short stretches of homology shared by *CAN1 *and *ALP1 *(or *LYP1*) [60], allowing them to anneal, followed by removal of the nonhomologous overhangs and ligation. Rad59-dependent, Rad51-independent interchromosomal translocation between *his3 *fragments was recently shown after induction of HO-breaks in the two recombining chromosomes [61]. Such an interchromosomal SSA event could also produce the types of rearrangements we have observed between *CAN1*, *LYP1 *and *ALP1*; however, the ends of chromosomes V and XIV not engaged in the SSA event would be left unrepaired and most likely would be lost after cell division unless the recombination event occurs in G2/M when sister-chromatids are present. Moreover, since we have shown that wildtype copies of *LYP1 *and *ALP1 *are still present in recombinants with *CAN1/LYP1/ALP1 *rearrangements, indicative of a nonreciprocal translocation event [60], and the parts of chromosome XIV that would be lost after SSA contain essential genes, SSA is unlikely to be the main recombination mechanism that gives rise to *CAN1*/*LYP1*/*ALP1 *rearrangements.

Besides SSA, BIR also matches the genetic requirements for *CAN1*/*LYP1*/*ALP1 *translocation formation. BIR is initiated by invasion of a duplex by a single-stranded 3'end of a one-sided DBS followed by replication to the chromosome end. Although Sgs1 has roles in recombination, specifically sister-chromatid exchange and resolution of recombination intermediates [2,9,62-64], it is not required for Rad51-independent BIR [57]. In contrast to SSA, the nonreciprocal nature of BIR events would maintain an intact copy of chromosome XIV in addition to the chromosome V/XIV translocation, suggesting that it is the more likely mechanism involved in *CAN1/LYP1/ALP1 *translocation. BIR has been implicated in the repair of one-sided DSBs, such as replication forks that collapsed at a single-strand break. BIR is also thought to allow telomerase-deficient cells (*tlc1Δ*), whose telomeres have shortened to a point where cells can no longer proliferate, to survive by extending what could be considered a one-sided DSB. Survivors can arise either by adding subtelomeric *Y' *elements in a Rad51-dependent mechanism (Type I) or by adding telomeric (G_1-3_T)_n _repeats in a Rad51-independent, but Rad59-dependent mechanism (Type II) [53-55]. The differential requirement for Rad51 and Rad59 in these two pathways is thought to result from the differences in length and sequence identity of the recombination substrates for Type I and Type II [53]. The long, nearly identical (~1% variation within the same strain) *Y' *elements [65] are thought to be better substrates for Rad51-mediated strand invasion, whereas Rad59 is able to use the shorter stretches of homology likely to be found within the highly variable (G_1-3_T)_n _repeats [53]. Besides BIR, evidence of homology-length dependency is also seen in gene conversion, with Rad59 becoming increasingly important as the length of sequence homology decreases [59]. This length-dependency may also explain our observation that *CAN1*/*LYP1*/*ALP1 *rearrangements, which show short regions of homology at the breakpoints [10,60], are inhibited by deletion of *RAD59*, but not by deletion of *RAD51*. Despite this differential effect on chromosome rearrangements between *CAN1*, *LYP1 *and *ALP1*, we observed no difference in the rate of overall genome instability between *sgs1Δ mec3Δ rad51Δ *and *sgs1Δ mec3Δ rad59Δ *mutants (P = 0.6892, Table 2), suggesting that the DNA lesions that give rise to viable GCRs are accessible to multiple repair pathways.

### Candidate screen reveals *EXO1 *as a strong suppressor of GCR formation in cells lacking Sgs1

To assess the possible role of other DNA metabolic factors in the suppression or formation of GCRs in cells lacking Sgs1, we introduced *exo1Δ*, *pol32Δ, rad1Δ, lig4Δ *and *yen1Δ *mutations into *sgs1Δ *and *sgs1Δ mec3Δ *mutants. Screening of the single, double and triple mutants revealed that *RAD1, POL32, LIG4 *and *YEN1 *are not strong suppressors of GCRs in wildtype cells, or in *sgs1Δ *or *sgs1Δ mec3Δ *mutants (Table 3). However, when we assessed the formation of *CAN1/LYP1/ALP1 *translocations in *sgs1Δ mec3Δ *mutants with *pol32Δ *or *rad1Δ *mutations we found that in both triple mutants *CAN1/LYP1/ALP1 *translocations were inhibited, revealing one *CAN1*/*LYP1 *translocation among 98 independent GCR clones in the *sgs1Δ mec3Δ pol32Δ *mutant and none (0/55) in the *sgs1Δ mec3Δ rad1Δ *mutant. Pol32, a nonessential subunit of polymerase δ that promotes processivity of the polymerase, is not required for SSA, but for DNA repair processes that involve extensive DNA synthesis, such as BIR [66], consistent with BIR being a pathway for *CAN1*/*LYP1*/*ALP1 *translocation formation. Although Rad1, a subunit of the Rad1-Rad10 nuclease critical for the removal of nonhomologous overhangs from annealed single strands in processes such as SSA [67,68], has not been shown to be required for BIR, it has been implicated in the removal of nonhomologous overhangs during GCR formation [69] and in recombination events that combine BIR and SSA processes [70,71].

**Table 3:** Effect of *lig4Δ, exo1Δ, rad1Δ, pol32 Δ *and *yen1 Δ *mutations on the accumulation of GCRs in checkpoint-proficient and checkpoint-deficient *sgs1 Δ *mutants

Relevant genotype*^a^*	GCR rate*^b^*	95% CI*^c^*
wildtype	1.1	< 1-6.2
*exo1*	24	7-79
*sgs1*	220	144-276
*sgs1 mec3 *	1297	1120-2030
*exo1 sgs1*	43800	30400-186000
*exo1 mec3 *	30	12-39
*exo1 mec3 sgs1 *	1168498	549530-3251000
*sgs1 mec3 exo1 lig4*	895988	701149-1236740
*lig4 *	16	ND
*sgs1 lig4*	80	35-254
*sgs1 mec3 lig4*	1335	948-2140
*yen1*	< 5	< 4-6
*sgs1 yen1*	81	57-265
*sgs1 mec3 yen1*	1089	254-2540
*pol32*	20	15-26
*sgs1 pol32*	25	< 24-105
*sgs1 mec3 pol32*	2317	1800-3110
*rad1*	10	< 9-23
*sgs1 rad1*	63	25-356
*sgs1 mec3 rad1*	1173	1020-1540

*^a ^*Strains with multiple gene deletions were constructed by sporulation of the appropriate heterozygous diploids. GCR rates with 95% confidence intervals (CIs) for wildtype [81], *sgs1 *[82], *sgs1 mec3 *[60] and *lig4 *[1] were reported previously and are included for comparison. Spores with both *sgs1Δ *and *pol32Δ *mutations grew very slowly and exhibited a low viable cell count on YPD in the GCR assay.

*^b ^*The rate of accumulating gross-chromosomal rearrangements (GCRs) is calculated by selecting for cells resistant to canavanine (Can^r^) and 5-fluoro-orotic acid (5-FOA^r^) and is expressed as Can^r ^5-FOA^r ^× 10^-10 ^[77].

*^c ^*95% confidence intervals (CI) for median GCR rates were calculated according to Nair [80].

Deletion of *EXO1*, coding for a nuclease with 5' to 3' exonuclease and flap-endonuclease activities, which has roles in mitotic and meiotic recombination as well as mutation avoidance and is thought to cooperate with Sgs1 in the processing of DSBs [19,72], induced the largest synergistic GCR rate increase we have observed to date in the *sgs1Δ *mutant. While *sgs1Δ *and *exo1Δ *single mutants exhibited moderately increased GCR rates compared to wildtype, the GCR rate of the *sgs1Δ exo1Δ *mutant was several hundred-fold higher than the rates of the single mutants (P < 0.0001, Table 3). This GCR rate increased another 26-fold upon deletion of *MEC3 *(P < 0.0001, Table 3). Screening of GCRs obtained from 66 independent cultures of the *sgs1Δ mec3Δ exo1Δ *mutant identified two *CAN1*/*LYP1*/*ALP1 *translocations, indicating a ~ 200-fold increase in the *CAN1*/*LYP1*/*ALP1 *translocation rate compared to the *sgs1Δ mec3Δ *mutant (3.5 × 10^-6 ^versus 1.7 × 10^-8^).

**Figure 1:**
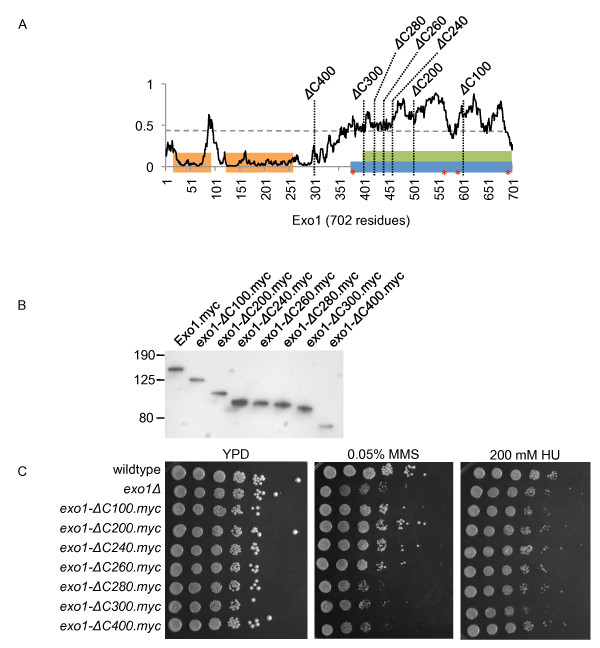
**Expression of C-terminal truncations of Exo1 and sensitivity to DNA-damaging agents**. **(A) **Intrinsic disorder prediction of Exo1 using the IUPred algorithm in which values above 0.5 indicate residues predicted to be intrinsically disordered and values below 0.5 to be ordered. The N-terminus, harboring conserved N- and I-nuclease domains, is predicted to be ordered, whereas the C-terminus, which appears devoid of enzymatic activity but contains phosphorylation sites and sites for interaction with mismatch repair proteins, is disordered. The sites at which the Exo1 truncations examined in this study terminate are indicted by vertical dotted lines. The location of conserved domains was adapted from reference [71]: nuclease domains (orange boxes, 16-96 aa, 123-257 aa), Mlh1 interaction domain (green box, 400-702 aa) and the Msh2 interaction domain (blue box, 368-702 aa). Phosphorylation sites at S372, S567, S587 and S692, implicated in checkpoint regulation [74], are indicated by red asterisks. **(B) **Western blot analysis of expression of myc-epitope tagged exo1 truncations and wildtype Exo1. Molecular weight markers (kD) are indicated on the left. **(C) **Cells expressing Exo1 truncations lacking 280 or more C-terminal residues are as sensitive to 0.05% MMS as the *exo1Δ *mutant whereas cells expressing exo1 truncations lacking 260 or fewer C-terminal residues show wildtype levels of resistance to MMS. No sensitivity to 200 mM hydroxyurea was observed for any of the tested yeast strains.

Exo1 contains conserved N-terminal N- and I-nuclease domains, apparently separated by a short disordered linker, and binding sites for the mismatch repair (MMR) proteins Mlh1 and Msh2 have been located within the C-terminal half of Exo1 [72-74], which is predicted to be intrinsically disordered (Figure 1A). Four phosphorylation sites (S372, S567, S587, S692) required for the regulation of the DNA-damage response have also been located in the disordered C-terminus [75]. To determine if the C-terminus of Exo1 plays a role in the suppression of genome instability in the *sgs1Δ *mutant we constructed a set of C-terminal deletions ranging from 100 to 400 residues (Figure 1A and 1B). We found that the C-terminal 260 residues of Exo1, making up 37% of the protein, play no major role in suppressing the accumulation of GCRs in the *sgs1Δ *mutant (Table 4). To test the possibility that the C-terminus with its binding sites for MMR proteins might be required for Exo1's role in mutation avoidance, but not for its role in suppressing GCRs, we utilized a fluctuation assay to determine the rate of accumulating canavanine resistance (Can^r^) mutations in strains expressing the various C-terminal Exo1 truncations (Table 5). As in the GCR assay, deletion of up to 260 residues had no effect on the Can^r ^mutation rate (P = 0.3524) whereas deletion of 280 or more residues caused a null phenotype (P = 0.0001). Similarly, only deletion of 280 or more residues caused sensitivity to methyl methanesulfonate (MMS) (Figure 1C). No sensitivity to 200 mM hydroxyurea was observed for any of the *exo1 *mutants (Figure 1C). Thus, deletion of up to 260 residues caused a phenotype similar to wildtype in all assays tested here, whereas deletion of 280 or more residues caused a null (*exo1Δ*) phenotype.

**Table 4:** Effect of *exo1 *mutations on the accumulation of GCRs in wildtype cells or cells lacking Sgs1 helicase.

Relevant genotype	GCR rate (Can^r ^5-FOA^r ^× 10^-10^)	95% CI*^a^* (Can^r ^5-FOA^r ^× 10^-10^)
*sgs1Δ*	89	57-177
*exo1Δ*	24	7-79
*exo1Δ sgs1Δ*	40484	31076-49848
*EXO1.myc *	5	4.4-5.3
*exo1-ΔC100.myc*	5	4-6
*exo1-ΔC200.myc*	< 4	< 3.8-4.8
*exo1-ΔC260.myc*	< 11	< 8-79
*exo1-ΔC280.myc*	< 11	< 8-29
*exo1-ΔC300.myc*	< 18	< 5-70
*exo1-ΔC400.myc*	13	5-41
*sgs1Δ EXO1.myc *	78	29-118
*sgs1Δ exo1-ΔC100.myc*	125	80-186
*sgs1Δ exo1-ΔC200.myc*	158	94-215
*sgs1Δ exo1-ΔC260.myc*	230	166-265
*sgs1Δ exo1-ΔC280.myc*	26840	22925-34036
*sgs1Δ exo1-ΔC300.myc*	31070	22871-33753
*sgs1Δ exo1-ΔC400.myc*	48190	39133-54471

*^a ^*95% confidence intervals (CI) for median GCR rates were calculated according to Nair [80].

**Table 5:** Effect of C-terminal deletions of Exo1 on the spontaneous mutation rate at the *CAN1 *locus.

Relevant genotype	*CAN1* (Can^r ^× 10^-7^)	95% CI*^a^* (Can^r ^× 10^-7^)	Increase over wildtype
wildtype	3.27	2.50 - 5.82	1
*exo1Δ*	11.47	10.10 - 28.52	3.5
*exo1-ΔC100.myc*	3.64	2.92 - 4.70	1.1
*exo1-ΔC200.myc*	5.31	3.90 - 5.90	1.6
*exo1-ΔC260.myc*	3.89	2.89 - 5.92	1.2
*exo1-ΔC280.myc*	8.37	6.94 - 16.18	2.6
*exo1-ΔC300.myc*	10.72	8.55 - 19.88	3.3
*exo1-ΔC400.myc*	13.16	9.06 - 18.19	4.0

*^a ^*95% confidence intervals (CI) for median Can^r ^rates were calculated according to Nair [80].

In addition to providing MMR protein interaction sites, the C-terminus of Exo1 contains four phosphorylation sites (S372, S567, S587, S692), which were recently shown to be important for the regulation of the DNA damage checkpoint in response to uncapped telomeres in a *cdc13-1 *mutant [75]. Unlike in a *cdc13-1 *mutant, we did not detect Exo1 phosphorylation in the *sgs1Δ *mutant (data not shown), and deletion of the C-terminal third of Exo1 (*exo1-ΔC260*), which contains three of the four phosphorylation sites (S567, S587, S692), had no effect on Exo1 function in the assays used here (Can^r ^mutation rate, GCR assay, MMS sensitivity). The fourth phosphorylation site (S372) is present in both the *exo1-ΔC260 *mutant and the *exo1-ΔC280 *mutant and, therefore, is not responsible for the different phenotypes associated with the two alleles. Thus, the known phosphorylation sites in Exo1 do not appear to be required for Exo1's role in mutation avoidance, resistance to MMS or suppression of GCRs in a *sgs1Δ *mutant. Instead, it is likely that the *ΔC280 *deletion affects Exo1 nuclease activity directly by disrupting intramolecular interactions with the N-terminus. The loss of yet unknown posttranslational modifications in this segment of Exo1 or an indirect effect caused by the loss of interaction with other cellular factors could also lead to the deficiency of the *exo1ΔC280 *allele.

Besides the overall increase in genome instability, *CAN1*/*LYP1*/*ALP1 *rearrangements seen in the *sgs1Δ mec3Δ *mutant were also present in the *sgs1Δ mec3Δ exo1Δ *mutant. Normally, Exo1 and Sgs1 function in independent end resection pathways that cooperate in the processing of DSBs, especially the long-range resection of the 5'-strand [19,20], and Marrero and Symington [21] recently showed that this extensive resection inhibits BIR in a plasmid-based assay. Besides upregulation of BIR, which was also accompanied by chromosome rearrangements, the *exo1Δ sgs1Δ *mutant was also more proficient in *de novo *telomere synthesis at HO-endonuclease-induced chromosome breaks [18,21]. The combination of increased BIR and more efficient *de novo *telomere addition, both of which have been identified as major mechanisms for the healing of chromosome V breaks in the GCR assay [76,77], likely also explains the remarkably strong accumulation of genome rearrangements originating from spontaneous DNA lesions in the *exo1Δ sgs1Δ *mutant studied here. Our study further adds that the *exo1Δ sgs1Δ *mutant has even greater potential for the accumulation of viable genome rearrangements, which is suppressed (~ 26-fold) in the *sgs1Δ exo1Δ *mutant by Mec3-dependent DNA-damage checkpoint functions (P < 0.0001). Nonhomologous endjoining does not appear to be a significant source for these genome rearrangements, as indicated by the lack of any effect of *LIG4 *gene deletion in mutants with various combinations of *sgs1Δ*, *exo1Δ *and *mec3Δ *mutations (e.g., GCR rate of *sgs1Δ mec3Δ exo1Δ *compared to *sgs1Δ mec3Δ exo1Δ lig4Δ*, P = 0.3953, Table 3); however, it is also plausible that in the absence of one repair pathway DNA lesions simply become substrates for various other available repair pathways.

## Conclusion

**Figure 2:**
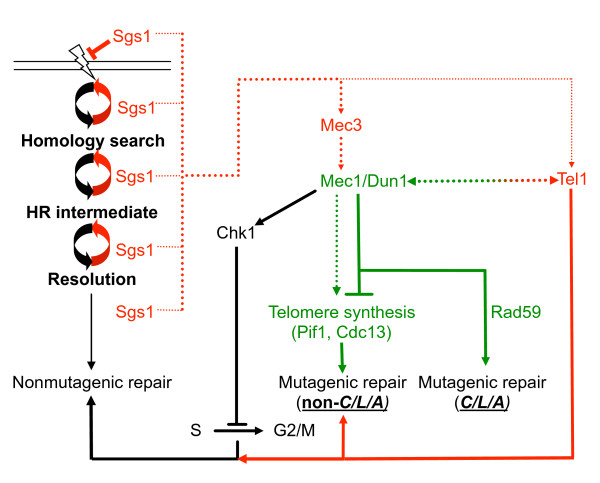
**Factors affecting the suppression and promotion of chromosomal translocations between short segments of homology in *CAN1*, *LYP1 *and *ALP1 *in cells lacking Sgs1**. In the absence of Sgs1, translocations between *CAN1, LYP1 *and *ALP1 *(referred to as *C/L/A*) are independently suppressed by the checkpoint components Mec3 and Tel1 (shown in red font), as suggested by the synergistic increases in the GCR rate and the *C/L/A *translocation rate of the *sgs1Δ *mutant upon deletion of *MEC3 *(*sgs1Δ mec3Δ*) and subsequently *TEL1 *(*sgs1Δ mec3Δ tel1Δ*). If Mec3 is absent (*sgs1Δ mec3Δ*), *C/L/A *translocations form through a pathway that requires Mec1, Dun1 and homologous recombination (HR) factors (shown in green font), especially Rad52 and Rad59. Mec1 most likely promotes translocations by inhibiting *de novo *telomere additions by regulating Pif1 and Cdc13. In addition to mutagenic repair that leads to *C/L/A *translocations, other types of mutagenic repair (e.g., translocations between other genes, *de novo *telomere additions, deletions, insertions, inversions) and most likely also nonmutagenic repair products are formed. If, in addition to Mec3, Tel1 is also absent (e.g., *sgs1Δ mec3Δ tel1Δ*), an even greater number of DNA lesions are channeled through the Mec1-dependent, *C/L/A*-promoting pathway. In contrast to *dun1Δ*, the *chk1Δ *mutation does not lead to a significant GCR rate increase in the *sgs1Δ mec3Δ *mutant and does not inhibit *C/L/A *translocation formation. Possibly, the inability to regulate cell cycle progression in the absence of Chk1 leads to increased formation of inviable GCRs. Dotted lines indicate events that occur in the absence of the protein from which the arrow originates; full lines indicate events that occur in the presence of the protein.

Our results indicate that spontaneous, interchromosomal translocations between short regions of sequence identity (5-41 bp), such as those present in the *CAN1*, *LYP1 *and *ALP1 *genes used in our assay, are promoted by Mec1/Dun1/Rad59-dependent pathways whereas Tel1, Mec3 and Sgs1 act as independent suppressors (Figure 2). The requirement for Pol32 and Rad1 in the translocation process further suggests the need for extensive DNA synthesis, such as seen in BIR, and the removal of nonhomologous overhangs from annealed single-strands, critical for SSA and implicated in GCR formation. Exo1 nuclease is a suppressor of overall genome rearrangements as well as *CAN1*/*LYP1*/*ALP1 *translocations when cells lack Sgs1 or both Sgs1 and Mec3. That the disordered, C-terminal third is dispensable for Exo1 function in our assays further indicates that physical interaction with MMR proteins in this region and regulation of Exo1 function in response to DNA-damage are not important for Exo1's role in the suppression of spontaneous GCRs, mutation avoidance and resistance to MMS.

## Methods

### Yeast strains and media

All strains used in this study are derived from KHSY802 (*MATa, ura3-52, trp1Δ63, his3Δ200, leu2Δ1, lys2Bgl, hom3-10, ade2Δ1, ade8, hxt13::URA3*) or the isogenic strain of the opposite mating type. Desired gene deletions were introduced by HR-mediated integration of PCR products containing a selectable marker cassette flanked by 50-nt sequences complementary to the target locus [78]. C-terminal truncations of Exo1 were constructed by replacing the desired DNA sequence at the chromosomal *EXO1 *locus with a myc-epitope encoding sequence amplified from pFA-13Myc.His3MX6 (a gift from Mark Longtine, Washington University, St. Louis). Expression of Exo1 truncation alleles was confirmed by Western blotting using monoclonal anti-c-myc antibody (Covance). All haploid strains with multiple gene deletions were obtained by sporulating diploids heterozygous for the desired mutations to minimize the risk of obtaining suppressors. This was especially important for combinations of mutations known to cause fitness defects, such as *sgs1Δ *and *pol32Δ*. Spore isolation was followed by genotyping of meiotic products by spotting on selective media or by PCR. All strains used in this study are listed in Table 6. Media for propagating yeast strains have been previously described [76,77].

**Table 6:** *Saccharomyces cerevisiae *strains used in this study

Strain ID	Genotype
KHSY802	*MATa, ura3-52, trp1Δ63, his3Δ200, leu2Δ1, lys2Bgl, hom3-10, ade2Δ1, ade8, hxt13::URA3*
RDKY 3721^a^	*MATa, ura3-52, trp1Δ63, his3Δ200, leu2Δ1, lys2Bgl, hom3-10, ade2Δ1, ade8, hxt13::URA3, rad17::HIS3*
RDKY 3739 ^a^	*MATa, ura3-52, trp1Δ63, his3Δ200, leu2Δ1, lys2Bgl, hom3-10, ade2Δ1, ade8, hxt13::URA3, dun1::HIS3*
RDKY 3745 ^a^	*MATa, ura3-52, trp1Δ63, his3Δ200, leu2Δ1, lys2Bgl, hom3-10, ade2Δ1, ade8, hxt13::URA3, chk1::HIS3*
RDKY 5209 ^a^	*MATa, ura3-52, trp1Δ63, his3Δ200, leu2Δ1, lys2Bgl, hom3-10, ade2Δ1, ade8, hxt13::URA3, tel1:G418*
KHSY 773	*MATa, ura3-52, trp1Δ63, his3Δ200, leu2Δ1, lys2Bgl, hom3-10, ade2Δ1, ade8, hxt13::URA3, sml1::TRP1, mec1::HIS3*
KHSY 884	*MATa, ura3-52, trp1Δ63, his3Δ200, leu2Δ1, lys2Bgl, hom3-10, ade2Δ1, ade8, hxt13::URA3, rad51::HIS3*
KHSY 906	*MATa, ura3-52, trp1Δ63, his3Δ200, leu2Δ1, lys2Bgl, hom3-10, ade2Δ1, ade8, hxt13::URA3, mec3::HIS3*
KHSY 1330	*MATa, ura3-52, trp1Δ63, his3Δ200, leu2Δ1, lys2Bgl, hom3-10, ade2Δ1, ade8, hxt13::URA3, sgs1::HIS3, mec1::TRP1, sml1::G418*
KHSY 1498	*MATa, ura3-52, trp1Δ63, his3Δ200, leu2Δ1, lys2Bgl, hom3-10, ade2Δ1, ade8, hxt13::URA3, sgs1::HIS3, mec1::TRP1, sml1::G418, mec3::G418*
KHSY 1524	*MATa, ura3-52, trp1Δ63, his3Δ200, leu2Δ1, lys2Bgl, hom3-10, ade2Δ1, ade8, hxt13::URA3, sgs1::TRP1*
KHSY 2260	*MATa, ura3-52, trp1Δ63, his3Δ200, leu2Δ1, lys2Bgl, hom3-10, ade2Δ1, ade8, hxt13::URA3, sgs1::TRP1, chk1::HIS3*
KHSY 2265	*MATa, ura3-52, trp1Δ63, his3Δ200, leu2Δ1, lys2Bgl, hom3-10, ade2Δ1, ade8, hxt13::URA3, sgs1::TRP1, rad17::HIS3*
KHSY 2280	*MATa, ura3-52, trp1Δ63, his3Δ200, leu2Δ1, lys2Bgl, hom3-10, ade2Δ1, ade8, hxt13::URA3, sgs1::TRP1, mec3::HIS3, rad59::G418*
KHSY 2283	*MATa, ura3-52, trp1Δ63, his3Δ200, leu2Δ1, lys2Bgl, hom3-10, ade2Δ1, ade8, hxt13::URA3, sgs1::TRP1, dun1::HIS3*
KHSY 2317	*MATa, ura3-52, trp1Δ63, his3Δ200, leu2Δ1, lys2Bgl, hom3-10, ade2Δ1, ade8, hxt13::URA3, tel1::G418, mec3::HIS3*
KHSY 2320	*MATa, ura3-52, trp1Δ63, his3Δ200, leu2Δ1, lys2Bgl, hom3-10, ade2Δ1, ade8, hxt13::URA3, sgs1::TRP1, mec3::HIS3*
KHSY 2330	*MATa, ura3-52, trp1Δ63, his3Δ200, leu2Δ1, lys2Bgl, hom3-10, ade2Δ1, ade8, hxt13::URA3, yen1::loxP-G418-loxP*
KHSY 2331	*MATa, ura3-52, trp1Δ63, his3Δ200, leu2Δ1, lys2Bgl, hom3-10, ade2Δ1, ade8, hxt13::URA3, lig4::loxP-G418-loxP*
KHSY 2336	*MATa, ura3-52, trp1Δ63, his3Δ200, leu2Δ1, lys2Bgl, hom3-10, ade2Δ1, ade8, hxt13::URA3, rad1::loxP-G418-loxP*
KHSY 2338	*MATa, ura3-52, trp1Δ63, his3Δ200, leu2Δ1, lys2Bgl, hom3-10, ade2Δ1, ade8, hxt13::URA3, exo1:loxp-G418-loxp*
KHSY 2388	*MATa, ura3-52, trp1Δ63, his3Δ200, leu2Δ1, lys2Bgl, hom3-10, ade2Δ1, ade8, hxt13::URA3, rad59::G418*
KHSY 2402	*MATa, ura3-52, trp1Δ63, his3Δ200, leu2Δ1, lys2Bgl, hom3-10, ade2Δ1, ade8, hxt13::URA3, sgs1::TRP1, exo1::loxP-G418-loxP*
KHSY 2408	*MATa, ura3-52, trp1Δ63, his3Δ200, leu2Δ1, lys2Bgl, hom3-10, ade2Δ1, ade8, hxt13::URA3, sgs1::TRP1*, *exo1::loxP-G418-loxP, mec3::HIS3*
KHSY 2424	*MATa, ura3-52, trp1Δ63, his3Δ200, leu2Δ1, lys2Bgl, hom3-10, ade2Δ1, ade8, hxt13::URA3, sgs1::TRP1*, *rad1::loxP-G418-loxP*
KHSY 2434	*MATa, ura3-52, trp1Δ63, his3Δ200, leu2Δ1, lys2Bgl, hom3-10, ade2Δ1, ade8, hxt13::URA3, sgs1::TRP1*, *rad1::loxP-G418-loxP, mec3::HIS3*
KHSY 2447	*MATa, ura3-52, trp1Δ63, his3Δ200, leu2Δ1, lys2Bgl, hom3-10, ade2Δ1, ade8, hxt13::URA3, sgs1::TRP1*, *lig4::loxP-G418-loxP*
KHSY 2448	*MATa, ura3-52, trp1Δ63, his3Δ200, leu2Δ1, lys2Bgl, hom3-10, ade2Δ1, ade8, hxt13::URA3, sgs1::TRP1*, *yen1::loxP-G418-loxP*
KHSY 2449	*MATa, ura3-52, trp1Δ63, his3Δ200, leu2Δ1, lys2Bgl, hom3-10, ade2Δ1, ade8, hxt13::URA3, sgs1::TRP1*, *yen1::loxP-G418-loxP, mec3::HIS3*
KHSY 2559	*MATa, ura3-52, trp1Δ63, his3Δ200, leu2Δ1, lys2Bgl, hom3-10, ade2Δ1, ade8, hxt13::URA3, mec3:: G418, rad17::HIS3*
KHSY 2565	*MATa, ura3-52, trp1Δ63, his3Δ200, leu2Δ1, lys2Bgl, hom3-10, ade2Δ1, ade8, hxt13::URA3, sgs1::TRP1, mec3::G418, rad17::HIS3 *
KHSY 2579	*MATa, ura3-52, trp1Δ63, his3Δ200, leu2Δ1, lys2Bgl, hom3-10, ade2Δ1, ade8, hxt13::URA3, sgs1:: TRP1, lig4::G418, mec3::HIS3 *
KHSY 2585	*MATa, ura3-52, trp1Δ63, his3Δ200, leu2Δ1, lys2Bgl, hom3-10, ade2Δ1, ade8, hxt13::URA3, sgs1::TRP1, tel1::G418, rad17::HIS3*
KHSY 2588	*MATa, ura3-52, trp1Δ63, his3Δ200, leu2Δ1, lys2Bgl, hom3-10, ade2Δ1, ade8, hxt13::URA3, tel1::G418, rad17::HIS3*
KHSY 2662	*MATa, ura3-52, trp1Δ63, his3Δ200, leu2Δ1, lys2Bgl, hom3-10, ade2Δ1, ade8, hxt13::URA3, sgs1::TRP1, mec3::HIS3, chk1::HIS3*
KHSY 2665	*MATa, ura3-52, trp1Δ63, his3Δ200, leu2Δ1, lys2Bgl, hom3-10, ade2Δ1, ade8, hxt13::URA3, sgs1::TRP1, mec3::HIS3, dun1::HIS3*
KHSY 2786	*MATa, ura3-52, trp1Δ63, his3Δ200, leu2Δ1, lys2Bgl, hom3-10, ade2Δ1, ade8, hxt13::URA3, sgs1::TRP1, exo1::loxP-G418-loxP, lig4::loxP-G418-loxP, mec3::HIS3 *
KHSY 3086	*MATa, ura3-52, trp1Δ63, his3Δ200, leu2Δ1, lys2Bgl, hom3-10, ade2Δ1, ade8, hxt13::URA3, sgs1::TRP1, mec3::G418, rad17::HIS3, tel1::HIS3*
KHSY 3223	*MATa, ura3-52, trp1Δ63, his3Δ200, leu2Δ1, lys2Bgl, hom3-10, ade2Δ1, ade8, hxt13::URA3, sgs1::TRP1, mec3::HIS3, tel1::G418*
KHSY 3231	*MATa, ura3-52, trp1Δ63, his3Δ200, leu2Δ1, lys2Bgl, hom3-10, ade2Δ1, ade8, hxt13::URA3, rad17::H1S3, mec3::HIS3, tel1::G418*
KHSY 3265	*MATa, ura3-52, trp1Δ63, his3Δ200, leu2Δ1, lys2Bgl, hom3-10, ade2Δ1, ade8, hxt13::URA3, exo1ΔC300.MYC.HIS*
KHSY 3271	*MATa, ura3-52, trp1Δ63, his3Δ200, leu2Δ1, lys2Bgl, hom3-10, ade2Δ1, ade8, hxt13::URA3, exo1ΔC400.MYC.HIS*
KHSY 3274	*MATa, ura3-52, trp1Δ63, his3Δ200, leu2Δ1, lys2Bgl, hom3-10, ade2Δ1, ade8, hxt13::URA3, exo1ΔC200.MYC.HIS, sgs1::TRP1*
KHSY 3278	*MATa, ura3-52, trp1Δ63, his3Δ200, leu2Δ1, lys2Bgl, hom3-10, ade2Δ1, ade8, hxt13::URA3, exo1ΔC100.MYC.HIS, sgs1::TRP1*
KHSY 3282	*MATa, ura3-52, trp1Δ63, his3Δ200, leu2Δ1, lys2Bgl, hom3-10, ade2Δ1, ade8, hxt13::URA3, exo1ΔC100.MYC.HIS*
KHSY 3287	*MATa, ura3-52, trp1Δ63, his3Δ200, leu2Δ1, lys2Bgl, hom3-10, ade2Δ1, ade8, hxt13::URA3, EXO1.MYC.HIS, sgs1::TRP1*
KHSY 3395	*MATa, ura3-52, trp1Δ63, his3Δ200, leu2Δ1, lys2Bgl, hom3-10, ade2Δ1, ade8, hxt13::URA3, EXO1.MYC.HIS*
KHSY 3396	*MATa, ura3-52, trp1Δ63, his3Δ200, leu2Δ1, lys2Bgl, hom3-10, ade2Δ1, ade8, hxt13::URA3, exo1Δ200.MYC.HIS*
KHSY 3402	*MATa, ura3-52, trp1Δ63, his3Δ200, leu2Δ1, lys2Bgl, hom3-10, ade2Δ1, ade8, hxt13::URA3, exo1Δ300.MYC.HIS, sgs1::TRP1*
KHSY 3635	*MATa, ura3-52, trp1Δ63, his3Δ200, leu2Δ1, lys2Bgl, hom3-10, ade2Δ1, ade8, hxt13::URA3, exo1::loxP-G418-loxP, mec3::HIS3 *
KHSY 3843	*MATa, ura3-52, trp1Δ63, his3Δ200, leu2Δ1, lys2Bgl, hom3-10, ade2Δ1, ade8, hxt13::URA3, exo1Δ400.MYC.HIS, sgs1::TRP1 *
KHSY 3849	*MATa, ura3-52, trp1Δ63, his3Δ200, leu2Δ1, lys2Bgl, hom3-10, ade2Δ1, ade8, hxt13::URA3, exo1Δ280.MYC.HIS *
KHSY 3857	*MATa, ura3-52, trp1Δ63, his3Δ200, leu2Δ1, lys2Bgl, hom3-10, ade2Δ1, ade8, hxt13::URA3, exo1Δ280.MYC.HIS, sgs1::TRP1 *
KHSY 3860	*MATa, ura3-52, trp1Δ63, his3Δ200, leu2Δ1, lys2Bgl, hom3-10, ade2Δ1, ade8, hxt13::URA3, exo1Δ260.MYC.HIS *
KHSY 3866	*MATa, ura3-52, trp1Δ63, his3Δ200, leu2Δ1, lys2Bgl, hom3-10, ade2Δ1, ade8, hxt13::URA3, exo1Δ240.MYC.HIS *
KHSY 3868	*MATa, ura3-52, trp1Δ63, his3Δ200, leu2Δ1, lys2Bgl, hom3-10, ade2Δ1, ade8, hxt13::URA3, exo1Δ260.MYC.HIS *
KHSY 3869	*MATa, ura3-52, trp1Δ63, his3Δ200, leu2Δ1, lys2Bgl, hom3-10, ade2Δ1, ade8, hxt13::URA3, exo1Δ260.MYC.HIS, sgs1::TRP1 *
KHSY 3875	*MATa, ura3-52, trp1Δ63, his3Δ200, leu2Δ1, lys2Bgl, hom3-10, ade2Δ1, ade8, hxt13::URA3, exo1Δ280.MYC.HIS, sgs1::TRP1 *

^a ^RDKY strains were a kind gift from Richard Kolodner (Ludwig Institute for Cancer Research, University of California - San Diego).

### Sensitivity to DNA damaging agents HU and MMS

Cell cultures were grown in yeast extract/peptone/dextrose (YPD) media and adjusted to OD600 = 1. Tenfold dilutions were spotted on YPD, YPD supplemented with 0.05% methyl-methanesulfonate (MMS) and YPD supplemented with 200 mM hydroxyurea (HU). Colony growth was documented after incubation at 30°C for 3 days.

### Fluctuation Assays

Rates of accumulating spontaneous gross-chromosomal rearrangements (GCRs) were determined by fluctuation analysis and the method of the median as previously described [77,79]. Cells with GCRs were detected by their resistance to canavanine and 5-fluoro-orotic acid (Can^r ^5-FOA^r^) due to simultaneous inactivation of the *CAN1 *and *URA3 *genes, both located within a 12 kb nonessential region on the left arm of chromosome V. The median GCR rate is reported with 95% confidence intervals [80]. GCR clones were screened by PCR to identify clones with rearrangements between *CAN1 *on chromosome V and *LYP1 *and/or *ALP1 *(collectively referred to as *CAN1*/*LYP1*/*ALP1 *rearrangements in the text), located in opposite orientations on the same arm of chromosome XIV [10]. To determine the rate of accumulating spontaneous mutations that lead to inactivation of the *CAN1 *gene, 3-ml YPD cultures expressing wildtype Exo1 or C-terminal truncations of Exo1were grown overnight and aliquots were plated on synthetic media lacking arginine (US Biological) supplemented with 240 mg ml^-1 ^canavanine (Sigma), and on YPD to obtain the viable cell count. Colonies were counted after two days of incubation at 30°C. At least twelve independent cultures from three isolates were analyzed per yeast strain. The median Can^r ^mutation rate is reported with 95% confidence intervals [80]. Statistical significance of differences in GCR rates was evaluated by using the Mann-Whitney test and programs from Dr. R. Lowry at Vassar College http://faculty.vassar.edu/lowry/VassarStats.html.

### Protein extraction and Western blot analysis

Cells were grown in YPD until they reached OD_600 _= 0.5. Whole cell extract was prepared from 5 ml of culture using a standard trichloroacetic acid (TCA) extraction. Briefly, cells were pelleted, vortexed with glass beads for 10 minutes in 200 μl of 20% TCA, followed by centrifugation for 2 minutes. The pellet was resuspended in sample buffer and pH was neutralized with 2 M Tris buffer (pH 7.6). Proteins were separated by PAGE, transferred to a PVDF membrane and incubated with monoclonal anti-c-myc antibody (Covance) to detect myc-tagged proteins. Bands were visualized using ECL Plus Chemiluminescence kit (GE Healthcare).

## List of abbreviations

BIR: break-induced replication; Can^r^: canavanine resistant; CI: confidence interval; DSB: double-strand break; 5-FOA^r^: 5-fluoro-orotic acid resistant; GCR: gross-chromosomal rearrangement; HR: homologous recombination; HU: hydroxyurea; MMR: mismatch repair; MMS: methyl methanesulfonate; SSA: single-strand annealing; TCA: trichloroacetic acid; YPD: yeast extract/peptone/dextrose.

## Competing interests

The authors declare that they have no competing interests.

## Authors' contributions

LD constructed yeast strains, performed experiments, analyzed data and performed statistical analyses. LH constructed yeast strains, performed experiments, analyzed data and performed statistical analyses. EV constructed yeast strains and performed experiments, KHS. designed the study, analyzed data and wrote the manuscript. All authors have read and approved the final manuscript.
